# Impact of Coenzyme Q10 Administration on Lead Acetate-Induced Testicular Damage in Rats

**DOI:** 10.1155/2020/4981386

**Published:** 2020-06-02

**Authors:** Manal El-khadragy, Wafa A. Al-Megrin, Norah A. AlSadhan, Dina M. Metwally, Rehab E. El-Hennamy, Fatma Elzahraa H. Salem, Rami B. Kassab, Ahmed E. Abdel Moneim

**Affiliations:** ^1^Biology Department, Faculty of Science, Princess Nourah Bint Abdulrahman University, Riyadh 84428, Saudi Arabia; ^2^Zoology and Entomology Department, Faculty of Science, Helwan University, Cairo 11795, Egypt; ^3^College of Medicine, AlMaarefa University, 11597 Riyadh, Saudi Arabia; ^4^Zoology Department, Faculty of Science, King Saud University, Riyadh 11451, Saudi Arabia; ^5^Faculty of Veterinary Medicine, Zagazig University, Zagazig, Egypt; ^6^Biology Department, Faculty of Science, Taibah University, Medina 42353, Saudi Arabia; ^7^Biology Department, Faculty of Science and Arts, Al Baha University, Almakhwah Branch, Saudi Arabia

## Abstract

Exposure to lead (Pb) causes multiorgan dysfunction including reproductive impairments. Here, we examined the protective effects of coenzyme Q10 (CoQ10) administration on testicular injury induced by lead acetate (PbAc) exposure in rats. This study employed four experimental groups (*n* = 7) that underwent seven days of treatment as follows: control group intraperitoneally (i.p.) treated with 0.1 ml of 0.9% NaCl containing 1% Tween 80 (*v* : *v*), CoQ10 group that was i.p. injected with 10 mg/kg CoQ10, PbAc group that was i.p. treated with PbAc (20 mg/kg), and PbAc+CoQ10 group that was i.p. injected with CoQ10 2 h after PbAc. PbAc injection resulted in increasing residual Pb levels in the testis and reducing testosterone, luteinizing hormone, and follicle-stimulating hormone levels. Additionally, PbAc exposure resulted in significant oxidative damage to the tissues on the testes. PbAc raised the levels of prooxidants (malondialdehyde and nitric oxide) and reduced the amount of endogenous antioxidative proteins (glutathione and its derivative enzymes, catalase, and superoxide dismutase) available in the cell. Moreover, PbAc induced the inflammatory response as evidenced by the upregulation of inflammatory mediators (tumor necrosis factor-alpha and interleukin-1 beta). Further, PbAc treatment induced apoptosis in the testicular cells, as indicated by an increase in Bax and caspase 3 expression, and reduced Bcl2 expression. CoQ10 supplementation improved testicular function by inhibiting Pb accumulation, oxidative stress, inflammation, cell death, and histopathological changes following PbAc exposure. Our findings suggest that CoQ10 can act as a natural therapeutic agent to protect against the reproductive impairments associated with PbAc exposure.

## 1. Introduction

Lead is an important environmental pollutant, which can cause serious illness if it is not controlled and monitored. It is widely used in industry, cosmetics, and medicine [1], and accumulation of lead in the environment can exert a toxic effect on humans and animals and causes damage to several important organs including the nervous system [2], skeleton [3], and cardiovascular system [4]. Recently, lead exposure has been highlighted as an important cause of testicular malfunction and male infertility [5, 6]. Excess exposure to lead products reduces semen quality and reproductive capacity causing male infertility [6]. It also promotes apoptosis and DNA damage in the spermatozoa [1] and activates Bax and caspase-3 in spermatogenic cells, which induces apoptosis [5]. It also impairs sperm function by reducing cyclic adenosine monophosphate and calcium levels of sperm and decreasing tyrosine phosphorylation of sperm proteins [7]. Pb exposure affects also the hormonal secretion as it suppresses the anterior pituitary secretion of LH and FSH [8]. Lead's toxic effect in the testes is facilitated by its ability to produce reactive oxygen species. It was reported that lead exposure causes an increase in membrane lipid peroxidation as well as reductions in the endogenous antioxidant [9]. It reduces the levels of glutathione, superoxide dismutase, and other enzymatic antioxidants [9].

Coenzyme Q10 (CoQ10) is concentrated in the mitochondria of eukaryotic cells, where it functions as a key element in the electron transport chain and thus an important factor in energy production. CoQ10 supplementation has been shown to have benefits in the treatment and prevention of a number of diseases including heart disease and cancer [10]. These benefits are associated with CoQ10's documented antioxidant and anti-inflammatory characteristics [11]. In addition, CoQ10 is able to prevent lipid peroxidation and adjust cytoplasmic redox potentials [12]. CoQ10 is found naturally in the seminal fluid and plays a central role in enhancing several key features of semen. Deficiency in CoQ10 has been linked to impaired sperm parameters [13]. Therefore, it has been used in several studies as a treatment for infertile men. It improves sperm density, sperm motility, sperm morphology, and sperm count [14, 15]. It also reduces the levels of follicle-stimulating hormone and luteinizing hormone [14, 15]. A recent study on men with idiopathic oligoasthenoteratozoospermia has proven its ability to increase sperm concentration and motility that is associated with the increase in the antioxidant capacity and enzymatic antioxidant activity as well [16]. Hence, this study was designed to evaluate the protective effect of CoQ10 supplementation following PbAc exposure in the testes of rats. The study will explore the antioxidant, antiapoptotic, and anti-inflammatory properties of CoQ10, with a specific emphasis on the *Nfe212*/*Hmox1* pathway.

## 2. Materials and Methods

### 2.1. Chemicals and Experimental Animals

Lead acetate trihydrate and CoQ10 were sourced from Sigma-Aldrich (St. Louis, MO, USA). The other reagents and chemicals were all analytical grade. Double-distilled water was used as the solvent. CoQ10 was dissolved in saline solution (0.9% NaCl) containing 1% Tween 80 (*v* : *v*) by stirring overnight at 25°C.

Male Wistar albino rats, aged ten weeks and weighing 150–170 g, were purchased from the animal facility of the Holding Company for Biological Products and Vaccines (Cairo, Egypt) and maintained in wire polypropylene cages under standard conditions (25 ± 2°C; 12 h light-dark cycle). The rats had access to water and standard rodent's diet *ad libitum*. Prior to the initialization of the experiment, rats were given one week for acclimatization. The experimental protocols were designed using the European Community Directive (86/609/EEC), and the national rules on animal care and all experiments were carried out in accordance with the NIH Guidelines for the Care and Use of Laboratory Animals 8^th^ edition. This study was approved by the Institutional Animal Ethics Committee for animal care and use at Helwan University (approval number: HU/Z/010-18).

### 2.2. Experimental Design

In order to explore the application of CoQ10 as a protective against lead acetate- (PbAc-) induced testicular toxicity, the rats were divided randomly into four groups (seven rats/group):

- Control group: rats were intraperitoneally (i.p.) treated with 0.1 ml of 0.9% NaCl containing 1% Tween 80 (*v* : *v*)
- CoQ10 group: CoQ10 was i.p. injected at 10 mg/kg according to the protocol described in a previous report [17]
- PbAc group: rats were i.p. treated with PbAc (20 mg/kg) based on the protocol developed by Moneim [18]
- PbAc+CoQ10 group: CoQ10 was i.p. injected 2 h after PbAc. All the treated groups were injected intraperitoneally for seven days

As reprotoxicity mediated by lead is well characterized, and several acute exposures at a much higher dosage as this have already been reported [19], we have applied this treatment regimen of seven days to mimic acute toxicity. CoQ10 and PbAc doses were selected based on the earlier published researches [17, 18].

### 2.3. Sampling and Tissue Preparations

At 24 h after the last injection, rats were sacrificed by using an overdose of isoflurane. Under anaesthesia and before sacrificing, blood was collected by syringe puncture of the abdominal aorta, and the serum was separated. The testes were removed. One of the testes from each rat was used for molecular and histopathological analyses, while the other was washed, weighed, and homogenized in ice-cold medium containing 50 mM Tris–HCl (pH 7.4). The homogenates were spun at 3000 × *g* at 4°C for 10 min; then, the supernatant was stored at –20°C for biochemical assay.

To evaluate the inflammatory and apoptotic markers using ELISA, testicular tissue was weighed and homogenized with ice-cold extracting buffer (0.1 ml), containing 0.1% nondenaturing detergent (Sigma, St. Louis, MO, USA) in phosphate-buffered saline with protease inhibitor cocktail (catalog number P8340, Sigma, St. Louis, MO, USA) in a 5 : 1 (*v* : *w*). After homogenization, the mixture was incubated for 10 min. The mixture was spun at 15,000 × *g* at 4°C (10 min). The developed supernatant was applied for the estimation of TNF-*α*, IL-1*β*, Bax, caspase 3, and Bcl-2.

### 2.4. Measurement of Lead Concentration in the Testis

The protocol established by Szkoda and Zmudzki [20] was used to estimate lead deposition in the left testis. Testicular specimens were dehydrated at 60°C and then dried at 150°C for 24 h in the oven. After which, the samples were dissolved in a hot solution of 1 M HNO_3_ then made up to 50 ml with deionized water. The samples were measured using a flame atomic absorption spectrophotometer (Perkin-Elmer, 3100) at 283.3 nm. Accumulated lead was expressed as *μ*g/g wet tissue weight.

### 2.5. Testicular Weight Assessment

The testicular absolute weight was measured using a sensitive weighing balance (Radwag, Model AS220/C/2, Clarkson Laboratory and Supply Inc., Chula Vista, CA, USA), whereas the relative testicular weight was calculated using the following formula:

(1) $$\begin{array}{c} \text{Relative testis weight}=\frac{\text{Left testis }\left(\text{LT}\right)}{\text{Body weight}}\times 100 \end{array}$$

### 2.6. Measurements of Sex Hormone Concentrations in the Blood

The estimation of testosterone, luteinizing hormone (LH), and follicle-stimulating hormone (FSH) levels was performed using specific commercial enzyme-linked immunosorbent assay (ELISA) kits (MyBioSource, Inc., San Diego, CA, USA) according to the manufacturer's instructions.

### 2.7. Determination of Redox Status in the Testis

Testicular nitric oxide (NO) content was determined using the optimized acid reduction method in the presence of nitrite. In this reaction, nitrous acid diazotized sulfanilamide couples with N-(1–naphthyl) ethylenediamine, resulting in the production of a bright reddish purple azo dye which can be quantified at 540 nm [21]. LPO in the testicular tissues was assessed using the malondialdehyde (MDA) method described by Ohkawa et al. [22]. Briefly, 100 mg of the testicular homogenate (pH 7.4) was blended with 100 *μ*l of sodium thioglycolate (1%), 100 *μ*l 100% trichloroacetic acid (TCA), and 250 *μ*l 1 N HCl. This mixture was then incubated for 20 min at 100°C and then centrifuged for 10 min (4000 rpm). The color was then used to estimate thiobarbituric acid reactive substance (TBARS) content and was evaluated at 532 nm. Additionally, the glutathione (GSH) levels in the testes was assessed via the reduction of 5,5′-dithiobis (2-nitrobenzoic acid) (Ellman's reagent) which turns yellow following reduction. The proportion of the reduced chromogen is directly proportional to the GSH concentration, and its absorbance can be evaluated at 405 nm [23].

### 2.8. Measurement of the Antioxidant Status of the Testes

The superoxide dismutase (SOD) activity was determined by measuring its ability to inhibit phenazine methosulfate-mediated reduction of nitroblue tetrazolium (NBT) [24]. Catalase (CAT) was estimated by adding 50 *μ*l of the testicular homogenate to 30 mM H_2_O_2_ in 50 mM potassium phosphate buffer (pH 8.0). H_2_O_2_ decomposition was then measured as a change in absorbance at 340 nm over 120 s at 20 s intervals [25]. The activity of glutathione reductase (GR) was assessed indirectly by assaying its catalysis of the glutathione reduction reaction in the presence of NADPH. NADPH oxidation to NADP^+^ was measured as a reduction in absorbance at 340 nm [26]. Finally, the activity of glutathione peroxidase (GPx) was estimated using the Paglia and Valentine [27] method. This assay measures the activity of GPx indirectly. The oxidized glutathione, resulting from the reduction of organic peroxide by GPx, is returned to its reduced state by glutathione reductase. This oxidizes NADPH to NADP^+^ which can be measured at 340 nm.

### 2.9. Inflammation Marker Assays

Tumor necrosis factor-*α* (TNF-*α*) and interleukin-1*β* (IL-1*β*) levels were evaluated in the testicular homogenate using ELISA kits purchased from Thermo Fisher Scientific. The kits were used as per the manufacturer's instructions.

### 2.10. Apoptotic Marker Assays

Testicular Bax, caspase 3, and Bcl-2 expressions were evaluated using commercial ELISA kits obtained from BioVision, Inc., Sigma-Aldrich, and Cusabio, respectively. These assays were completed as per the manufacturer s' instructions.

### 2.11. Quantitative Real Time PCR

The RNeasy Plus Minikit (Qiagen, Valencia, CA) was used to purify the total RNA from the testicular tissues. cDNA was prepared using the RevertAid™ H Minus Reverse Transcriptase kit (Fermentas, Thermo Fisher Scientific Inc., Canada). For real-time PCR analysis, samples of cDNA were run in triplicate. The reaction was performed using the Power SYBR® Green Mastermix (Life Technologies, CA) on an Applied Biosystems 7500 system. The PCR cycling thermal conditions were established as follows: preliminary denaturation at 95°C for 12 min, then by 45 cycles of denaturation at 94°C for 60 s and annealing at 55°C for 60s, extension at 72°C for 90 s, and afterwards held for a final extension at 72°C for 10 min. Gene expression values were normalized against *Actb*. The primer sequences and accession numbers for each of these genes are provided in Table 1.

### 2.12. Histological Examinations

The testes were fixed in 10% neutral buffered formalin for 24 h at room temperature. The tissues were dehydrated using an ascending series of alcohol concentrations, cleared in xylene, and embedded in paraffin wax. Paraffin blocks were sectioned at 5 *μ*m thickness then hydrated and stained with hematoxylin and eosin according to the Drury and Wallington [28] method. Sections were examined using a Nikon microscope (Eclipse E200-LED, Tokyo, Japan). Images represent the 400x magnification.

### 2.13. Immunohistochemical Investigations

After the deparaffinization and hydration of the testis sections, the peroxidase activity was blocked by H_2_O_2_ (3%). The sections were rinsed twice with distilled water, followed by phosphate buffer for 5 min. After the washing procedure, the sections were incubated with a blocking solution, the bovine serum albumin (5%), for 60 min. Testis sections were incubated with primary antibodies for caspase 3 (Santa Cruz, CA, USA) at 4°C overnight. Washing the sections with PBS was applied three times (5 min) the next day. Then, the sections were incubated with the secondary antibodies for 30 min. Another wash with PBS for three times (5 min) was performed before the incubation with diaminobenzidine for 5 min. Finally, sections were washed with distilled water (5 min) then stained with hematoxylin. Sections were investigated with the Eclipse E200-LED microscope (Nikon, Kawasaki, Japan) at the magnification of 400x.

### 2.14. Statistical Analysis

The results are expressed as the mean ± standard error of the mean (SEM). Data were analyzed by one-way analysis of variance (ANOVA) using the statistical package SPSS, version 17. In order to compare the significance between groups, Duncan's test was used post hoc. A probability level of *p* < 0.05 was considered statistically significant.

## 3. Results

**Figure 1:**
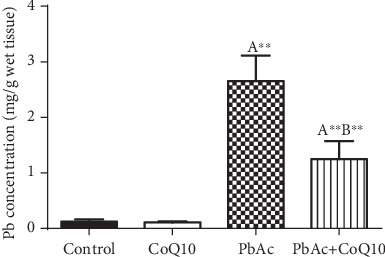
Effects of coenzyme Q10 (CoQ10, 10 mg/kg) administration on the concentration of Pb in the testis of rats treated with lead acetate (PbAc, 20 mg/kg). Data was represented as mean ± SEM (*n* = 7). ^A,B^Significant change at *p* < 0.05; ^∗^^,^^∗∗^Significant variation at *p* < 0.01 and *p* < 0.001, respectively, as compared to the control and PbAc groups, respectively. Data was analyzed by one-way ANOVA using Duncan's post hoc test.

Lead accumulates in various body tissues including the reproductive organs. Here, PbAc i.p. injection (20 mg/kg, seven days) significantly increased Pb concentrations (*p* < 0.001) in testicular homogenates when compared to the vehicle control group. On the other hand, rats posttreated with CoQ10 showed a marked decrease in Pb levels in the testicular tissues when compared to the PbAc-treated group (Figure 1).

**Figure 2:**
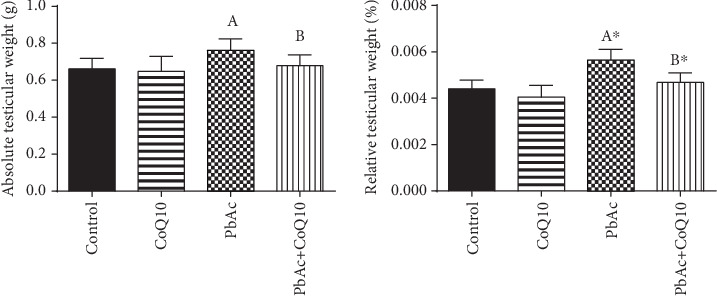
Effects of coenzyme Q10 (CoQ10, 10 mg/kg) administration on the absolute and relative testicular weight in rats treated with lead acetate (PbAc, 20 mg/kg). All data represented as mean ± SEM (*n* = 7). ^A,B^Significant change at *p* < 0.05; ∗Significant variation at *p* < 0.01 as compared to the control and PbAc groups, respectively. Data was analyzed by one-way ANOVA using Duncan's post hoc test.

Data in Figure 2 shows that Pb accumulation correlates with the increased absolute and relative testicular weights in the lead-treated groups when compared to the control group. This weight gain decreased significantly (*p* < 0.001) following CoQ10 treatment.

**Figure 3:**
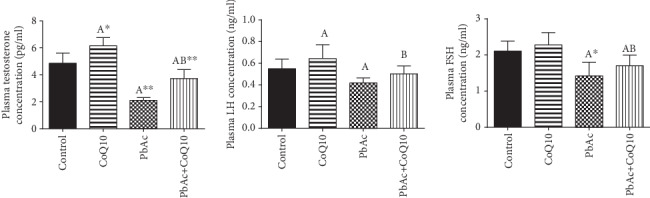
Effects of coenzyme Q10 (CoQ10, 10 mg/kg) administration on the plasma levels of testosterone, LH, and FSH in rats treated with lead acetate (PbAc, 20 mg/kg). All data represented as mean ± SEM (*n* = 7). ^A,B^Significant change at *p* < 0.05; ^∗^^,^^∗∗^Significant variation at *p* < 0.01 and *p* < 0.001, respectively, as compared to the control and PbAc groups, respectively. Data was analyzed by one-way ANOVA using Duncan's post hoc test.

In order to evaluate PbAc-induced reprotoxicity, serological levels of the sex hormones were determined (Figure 3). PbAc injection significantly decreased the levels of testosterone (*p* < 0.001), LH (*p* < 0.05), and FSH (*p* < 0.01) when compared to the control. In contrast, CoQ10 supplementation of Pb-free rats showed a significant increase in testosterone and LH levels, while FSH remained unchanged when compared to the control group. Interestingly, the group treated with both PbAc and CoQ10 showed a significant increase in the levels of these sex hormones when compared to the PbAc group.

**Figure 4:**
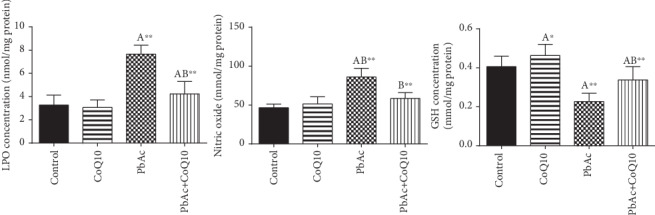
Effects of coenzyme Q10 (CoQ10, 10 mg/kg) administration on lipid peroxidation (LPO), nitric oxide (NO), and reduced glutathione (GSH) concentrations in the testis of rats treated with lead acetate (PbAc, 20 mg/kg). Data was represented as mean ± SEM (*n* = 7). ^A,B^Significant change at *p* < 0.05; ^∗^^,^^∗∗^Significant variation at *p* < 0.01 and *p* < 0.001, respectively, as compared to the control and PbAc groups, respectively. Data was analyzed by one-way ANOVA using Duncan's post hoc test.

**Figure 5:**
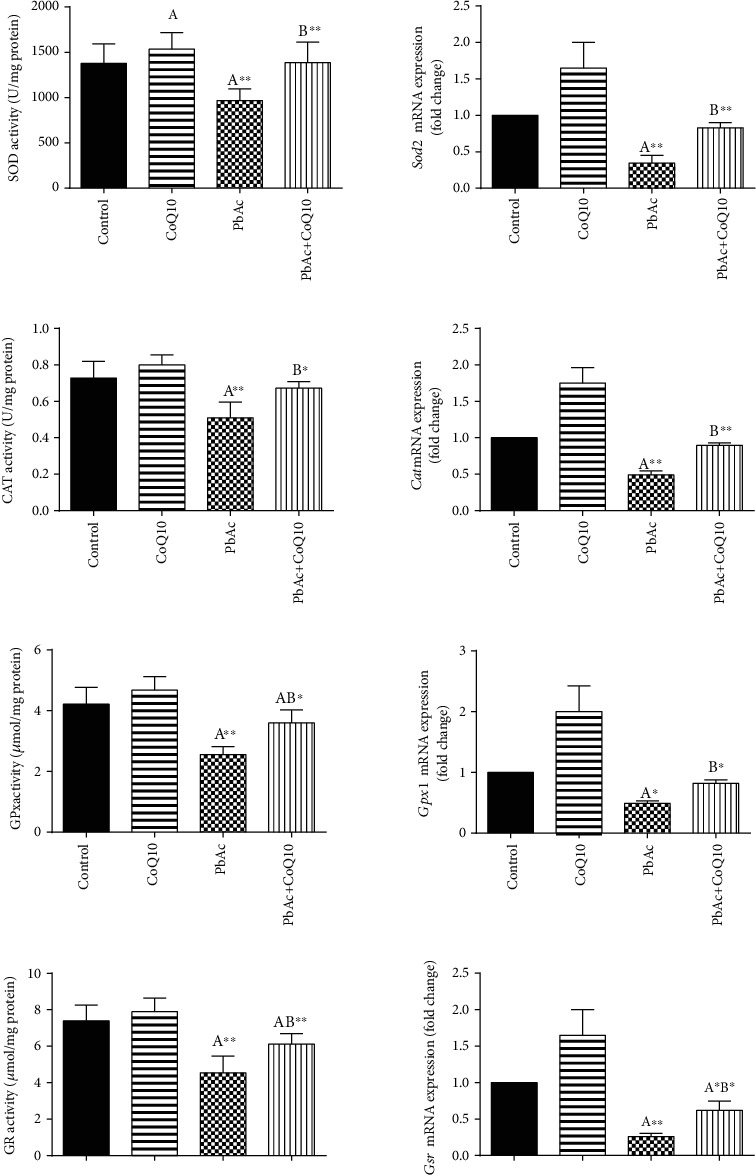
Effects of coenzyme Q10 (CoQ10, 10 mg/kg) administration on the activity of superoxide dismutase (SOD), catalase (CAT), glutathione peroxidase (GPx), glutathione reductase (GR), and their corresponding mRNA expressions in the testis of rats exposed to lead acetate (PbAc, 20 mg/kg). Data of the antioxidant enzyme activity was represented as mean ± SEM (*n* = 7), while the values of mRNA expressions (mean ± SEM of triplicate assays) were normalized to the *Actb* mRNA levels and are expressed as the fold change relative to the control group. ^A,B^Significant change at *p* < 0.05; ^∗^^,^^∗∗^Significant variation at *p* < 0.01 and *p* < 0.001, respectively, as compared to the control and PbAc groups, respectively. Data was analyzed by one-way ANOVA using Duncan's post hoc test.

PbAc injection impaired the balance between pro- and antioxidants in the testicular tissues as demonstrated by the increased LPO (*p* < 0.001) and NO (*p* < 0.001) concentrations and increased glutathione depletion (*p* < 0.001) observed in PbAc-treated rats (Figure 4). Moreover, we were able to demonstrate a significant decrease in the activity and expression of the antioxidant enzymes ((GPx1, *Gpx1*), (GR, *Gsr*), (CAT, *Cat*), and (SOD, *Sod2*)) following PbAc injection when compared to the control group. As expected, CoQ10 supplementation restored the redox status by decreasing the prooxidant concentration and promoting the activity of the endogenous antioxidant enzymes and their expression as demonstrated by changes in these parameters when compared to the PbAc-treated group (Figure 5).

**Figure 6:**
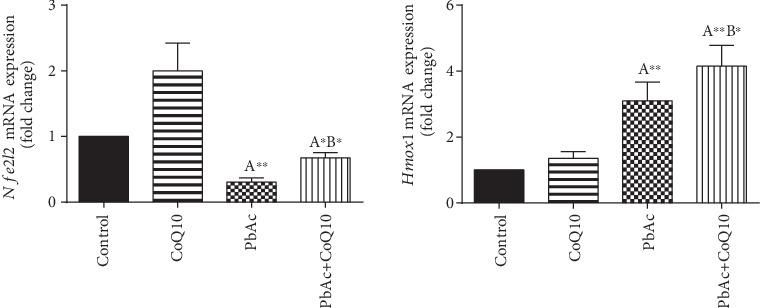
Effects of coenzyme Q10 (CoQ10, 10 mg/kg) administration on the mRNA levels of *Nfe212* and *Hmox1* in the testes of rats exposed to lead acetate (PbAc, 20 mg/kg). Data was represented as mean ± SEM of triplicate assays. Data was first normalized to the *Actb* mRNA levels then was expressed as a fold change relative to the control group. ^A,B^Significant change at *p* < 0.05; ^∗^^,^^∗∗^Significant variation at *p* < 0.01 and *p* < 0.001, respectively, as compared to the control and PbAc groups, respectively.

To understand the molecular antioxidant activity of CoQ10, testicular expression of *Nfe2l2* and *Hmox1* was evaluated. Nrf2 regulates the expression and activity of numerous antioxidant and detoxifying proteins. PbAc treatment significantly downregulated (*p* < 0.001) *Nfe2l2* expression, while upregulated (*p* < 0.001) *Hmox1* expression when compared to the control group. When CoQ10 supplementation was added after PbAc treatment, both *Nfe2l2* and *Hmox1* expressions were upregulated when compared to PbAc treatment (Figure 6).

**Figure 7:**
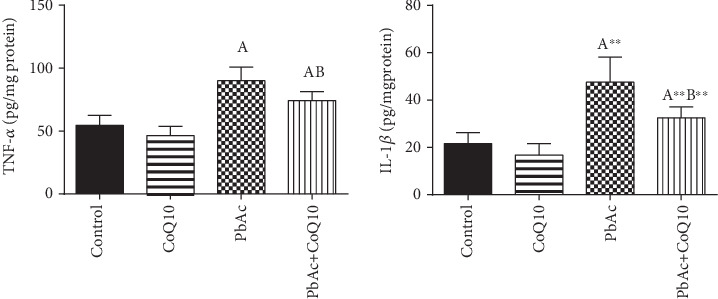
Effects of coenzyme Q10 (CoQ10, 10 mg/kg) administration on the levels of tumor necrosis factor-*α* (TNF-*α*) and interleukin-1*β* (IL-1*β*) in the testis of rats treated with lead acetate (PbAc, 20 mg/kg). Data was represented as mean ± SEM (*n* = 7). ^A,B^Significant change at *p* < 0.05; ^∗^^,^^∗∗^Significant variation at *p* < 0.01 and *p* < 0.001, respectively, as compared to the control and PbAc groups, respectively.

Inflammation has been suggested as one of the pathways that affect reproductive function following heavy metal intoxication. Rats injected with PbAc showed increased inflammation in the testicular tissues as demonstrated by increases in TNF-*α* (*p* < 0.05) and IL-1*β* (*p* < 0.001) expression when compared to the control group, while CoQ10 posttreatment attenuated this response following PbAc exposure (Figure 7).

**Figure 8:**
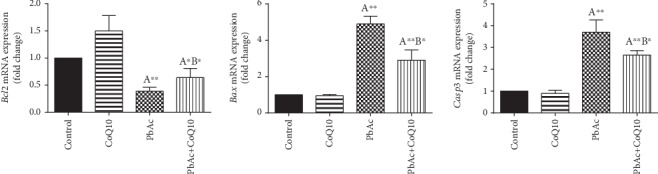
Effects of coenzyme Q10 (CoQ10, 10 mg/kg) administration on the mRNA expression of *Bcl2*, *Bax*, and *Casp3* in the testes of rats exposed to lead acetate (PbAc, 20 mg/kg). Data was represented as mean ± SEM of triplicate assays. Data was first normalized to the *Actb* mRNA levels then was expressed as the fold change relative to the control group. ^A,B^Significant change at *p* < 0.05; ^∗,∗∗^Significant variation at *p* < 0.01 and *p* < 0.001, respectively, as compared to the control and PbAc groups, respectively. Data was analyzed by one-way ANOVA using Duncan's post hoc test.

**Figure 9:**
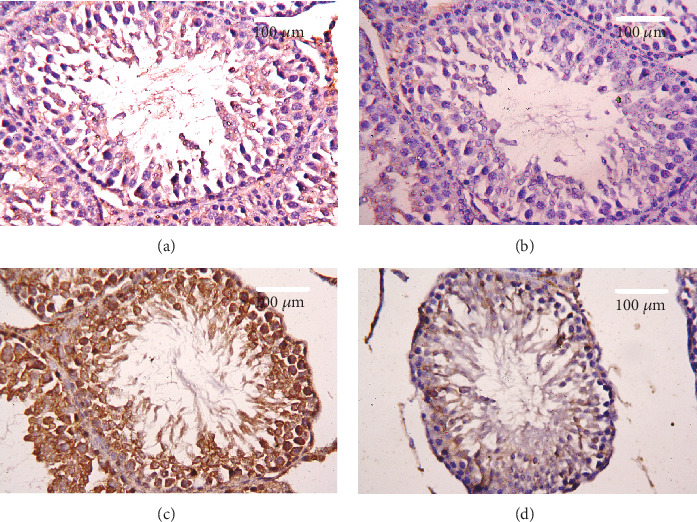
Effects of coenzyme Q10 (CoQ10, 10 mg/kg) administration on the caspase-3 expression in the testis tissue after lead acetate (PbAc, 20 mg/kg) exposure. (a) Control group, (b) CoQ10-administered group, (c) PbAc-exposed group, and (d) PbAc+CoQ10-treated group. Scale bar = 100 *μ*m.

To evaluate cell death and the apoptotic cascade in the testicular tissue, the proapoptotic markers (Bax and caspase 3) and the antiapoptotic marker (Bcl2) were evaluated in each of the various groups. Rats treated with PbAc exhibited testicular cell loss as evidenced by the increased expression of *Bax* (*p* < 0.001) and *Casp3* (*p* < 0.001) and decreased *Bcl2* (*p* < 0.001) expression when compared to the control group, while CoQ10 injection after PbAc exposure was found to protect the testicular tissues by enhancing *Bcl2* expression and inhibiting *Bax* and *Casp3* expression (Figure 8). Constant with the gene expression level of caspase-3, immunohistochemical analysis obviously showed that PbAc enhances the apoptotic cascade in the testicular tissue as presented by elevating the immunostaining intensity for the proapoptotic protein, caspase-3. However, postadministration of CoQ10 markedly decreased the number of the positively stained spermatogenic epithelial cells for caspase-3 immunoreactivity (Figure 9).

**Figure 10:**
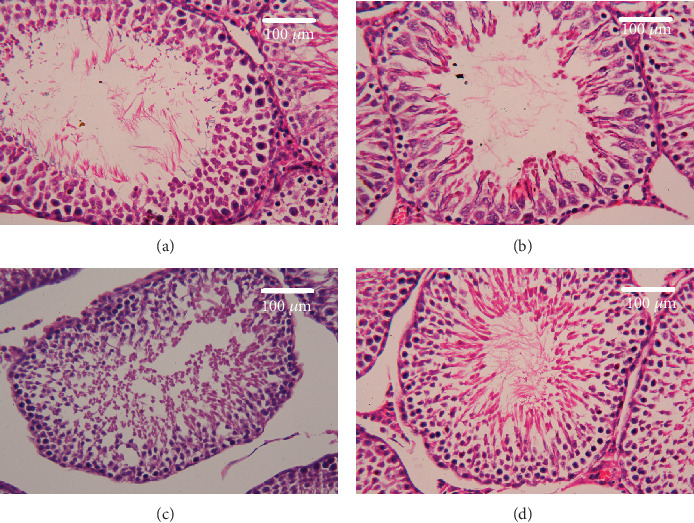
Effects of coenzyme Q10 (CoQ10, 10 mg/kg) administration on the histopathological changes in the testis tissue after lead acetate (PbAc, 20 mg/kg) exposure. (a) Control group, (b) CoQ10-administered group, (c) PbAc-exposed group, and (d) PbAc+CoQ10-treated group. Scale bar = 100 *μ*m.

Histological examination of testicular sections stained with H&E was represented in Figure 10. Control and CoQ10-treated alone rats revealed normal testicular morphology with typical and functional seminiferous tubules with well-arranged spermatogenic cells displaying all stages and the interstitial cells filling the space between the seminiferous tubules (Figures 10(a) and 10(b), respectively). On the other hand, PbAc-injected rats exhibited deleterious histopathological alterations verified by degeneration of spermatogenic cells, detachment of the spermatogenic epithelial cells from the basement membrane, and appearance of a vacuolated area within the seminiferous tubules (Figure 10(c)). Interestingly, CoQ10 posttreatment repaired these abnormalities and largely preserved the testicular structures (Figure 10(d)).

## 4. Discussion

Pb toxicity primarily affects the testes [5, 29]. Testicular toxicity induced by PbAc treatment was characterized by diminished plasma testosterone and LH and FSH concentrations, which was in agreement with the observations of Doumouchtsis et al. [30] and Soleimanzadeh *et al*. [31]. Hormonal reduction resulting from Pb exposure is attributed to impairment of the hypothalamus-pituitary-gonadal axis [8, 30, 32]. Pb exposure causes degeneration of the pituitary gland gonadotrophic cells [33] and induces apoptotic signals in the Leydig cells [7]. In addition, Pb inhibits steroidogenic enzyme production in Leydig cells resulting in reduced testosterone secretion [34].

The high levels of polyunsaturated fatty acids in the testicular cells make them vulnerable to oxidative stress [35]. Consequently, Pb toxicity-induced testicular malfunction may result from its ability to evoke membrane lipid peroxidation promoting oxidative stress and apoptosis [5, 36, 37]. Likewise, the prooxidant parameters, LPO and NO, increased after Pb injection. On the other hand, the antioxidant parameters were diminished with Pb treatment. The gene expression of the antioxidant enzymes, Cat mRNA, Gpx1 mRNA, Gsr mRNA, and Sod mRNA, and its corresponding proteins (CAT, GPX, GR, and SOD) were downregulated after Pb exposure. The GSH level and the activity of the antioxidant enzymes (GPx, GR, CAT, and SOD) were also all reduced. This depletion may be caused by Pb binding of the metal cofactors of these enzymes or from the interaction of Pb and their SH group, reducing overall function [38].

Moreover, the apoptotic pathway was activated by Pb administration which is demonstrated by the proapoptotic genes (Bax and caspase-3) upregulation, as well as the downregulation of the antiapoptotic gene, Bcl2. Similarly, previous studies revealed a testicular damage and apoptosis in the spermatogenic cells with Pb exposure [5, 7].

The inflammatory responses were elevated in the testes after Pb exposure, which was evidenced by the rise in the proinflammatory cytokines, TNF-*α* and IL1-*β*. Salama et al. [39] also reported an elevation in the proinflammatory cytokine levels in the kidneys of rats after Pb exposure. Furthermore, PbAc administration resulted in the downregulation of *Nfe212* gene expression. Suppression of *Nfe212* induces apoptosis, activates the inflammatory response, and decreases expression of the antioxidant enzymes [40]. *Hmox1* gene expression was upregulated. The activation of the *Hmox1* pathway may act to counter the elevation in the ROS and the inflammatory response [41].

Coenzyme Q10 is a natural antioxidant that plays a fundamental role in the electron transport chain [42]. It has been shown to exhibit a protective effect on the testes when they are exposed to magnetic fields [43], ischemia/reperfusion injuries [44], and sodium arsenite toxicity [45]. Here, we focused on its effect after PbAc exposure and we found that it could improve testicular function even in the presence of Pb. The absolute and relative testicular weight was restored following CoQ10 supplementation, while the testosterone, LH, and FSH levels were improved; signs of oxidative stress were decreased as evidenced by the reduction in LPO and NO concentrations. In addition, CoQ10 enhanced expression and level of glutathione and the antioxidant enzymes, CAT, SOD, GPx, and GR. Fouad et al. [45] revealed that CoQ10 could suppress oxidative stress in the testis by inhibiting lipid peroxidation and enhancing antioxidant enzyme activity. This in turn can counteract oxidative damage and sustain the function of Leydig cells protecting testosterone secretion [46]. The key application of CoQ10 in the testis is to increase the levels of CoQ10 and its reduced form, ubiquinol, in the semen [13]. Ubiquinol is a potent fat-soluble antioxidant that can regenerate other antioxidants including vitamins E and C [47]. It also eliminates peroxyl radicals resulting from the lipid peroxidation process [48].

The antiapoptotic effect of CoQ10 has been described by several studies [40, 49], and it was confirmed in this study when CoQ10 treatment upregulated the expression of the antiapoptotic gene *Bcl2* and downregulated proapoptotic genes *Casp3* and *Bax*. Papucci *et al*. [50] attributed the antiapoptotic effect of CoQ10 to the inhibition of DNA fragmentation and mitochondrial depolarization as well as increasing ATP levels. Additionally, CoQ10 inhibits nuclear translocation of apoptosis-inducing factors and prevents cell death via the inhibition of mitochondrial complex I activity [51].

CoQ10 is implicated in the prevention of inflammation in the liver [52] and kidneys [40]. This anti-inflammatory effect can now be extended to the testes, as CoQ10 administration following PbAc reduced the concentrations of proinflammatory cytokines, TNF-*α* and IL-1*β*, consequently attenuating inflammation. Meanwhile, it activated the *Nfe212/Hmox1* pathway by upregulating both *Nfe212* and *Hmox1* gene expressions. Previous studies indicated that CoQ10 exerts its cytoprotective role and antioxidant activity by activating the *Nfe212/Hmox1* pathway [53]. *Nfe212/Hmox1* signaling induces the transcription of the cytoprotective and antioxidant enzymes [54] and protects cells from damage and death [55]. *Nfe212*, the transcription factor, translocates from cytosol into the nucleus and bind to the antioxidant response element so upregulating the genes encoding antioxidant enzymes [56]. In addition, *Nfe212* activation resulted in the inhibition of NF-*κ*B and in turn reduces the proinflammatory cytokines [57]. Increased *Hmox1* activity generates two important metabolites, bilirubin and CO. Bilirubin acts as an antioxidant that inhibits lipid peroxidation [58], while CO protects the cell during oxidative stress [59] thus inhibiting apoptosis in various cells [53], and attenuates the production of proinflammatory cytokine [57].

## 5. Conclusion

Taken together, we can conclude that CoQ10 posttreatment ameliorates Pb-induced testicular toxicity by reducing oxidative stress, apoptosis, and inflammation. These observations may be the result of *Nfe212/Hmox1* pathway activation, but this will need further study for confirmation.

## Figures and Tables

**Table 1:** Primer sequences of genes analyzed by real time PCR.

Name	Sense (5′—3′)	Antisense (5′—3′)
*Actb*	GGCATCCTGACCCTGAAGTA	GGGGTGTTGAAGGTCTCAAA
*Sod2*	AGCTGCACCACAGCAAGCAC	TCCACCACCCTTAGGGCTCA
*Cat*	TCCGGGATCTTTTTAACGCCATTG	TCGAGCACGGTAGGGACAGTTCAC
*Gpx1*	CGGTTTCCCGTGCAATCAGT	ACACCGGGGACCAAATGATG
*Gsr*	TGCACTTCCCGGTAGGAAAC	GATCGCAACTGGGGTGAGAA
*Nfe2l2*	GGTTGCCCACATTCCCAAAC	GGCTGGGAATATCCAGGGC
*Hmox1*	GCGAAACAAGCAGAACCCA	GCTCAGGATGAGTACCTCCCA
*Nos2*	CGAAACGCTTCACTTCCAA	TGAGCCTATATTGCTGTGGCT
*Il1b*	TGCCACCTTTTGACAGTGATG	TTCTTGTGACCCTGAGCGAC
*Tnf-α*	AGAGGCACTCCCCCAAAAGA	CGATCACCCCGAAGTTCAGT
*Bcl2*	GACAGAAGATCATGCCGTCC	GGTACCAATGGCACTTCAAG
*Bax*	CTGAGCTGACCTTGGAGC	GACTCCAGCCACAAAGATG
*Casp3*	GAGCTTGGAACGGTACGCTA	CCGTACCAGAGCGAGATGAC

Abbreviations of the genes: *Actb*: beta actin; *Sod2:* superoxide dismutase 2 mitochondrial (MnSOD); *Cat*: catalase; *Gpx1*: glutathione peroxidase 1; *Gsr*: glutathione reductase; *Nfe2l2*: nuclear factor erythroid 2-related factor 2; *Hmox1*: heme oxygenase-1; *Nos2*: inducible nitric oxide synthase; *Il-1β*: interleukin 1-beta; *Tnf-α*: tumor necrosis factor-alpha; *Bcl2*: B-cell lymphoma 2; *Bax*: Bcl-2-associated X protein; *Casp3*: caspase 3.

## Data Availability

All relevant data are within the paper.
